# The broad-spectrum activity of perampanel: state of the art and future perspective of AMPA antagonism beyond epilepsy

**DOI:** 10.3389/fneur.2023.1182304

**Published:** 2023-07-06

**Authors:** Fabio Perversi, Cinzia Costa, Angelo Labate, Simona Lattanzi, Claudio Liguori, Marta Maschio, Stefano Meletti, Lino Nobili, Francesca Felicia Operto, Andrea Romigi, Emilio Russo, Carlo Di Bonaventura

**Affiliations:** ^1^Polistudium s.r.l., Milan, Italy; ^2^Section of Neurology, Department of Medicine and Surgery, University of Perugia, Perugia, Italy; ^3^Neurological Clinic, S. Maria Della Misericordia Hospital, Perugia, Italy; ^4^Neurophysiopatology and Movement Disorders Clinic, University of Messina, Messina, Italy; ^5^Neurological Clinic, Department of Experimental and Clinical Medicine, Marche Polytechnic University, Ancona, Italy; ^6^Department of Systems Medicine, University of Rome ‘Tor Vergata”, Rome, Italy; ^7^Epilepsy Center, Neurology Unit, University Hospital “Tor Vergata”, Rome, Italy; ^8^Center for Tumor-Related Epilepsy, UOSD Neuro-Oncology, IRCCS Regina Elena National Cancer Institute, Rome, Italy; ^9^Neurology Department, University Hospital of Modena, Modena, Italy; ^10^Department of Biomedical, Metabolic, and Neural Sciences, University of Modena and Reggio-Emilia, Modena, Italy; ^11^Child Neuropsychiatry Unit, IRCCS Istituto G. Gaslini, Genova, Italy; ^12^Department of Neuroscience, Rehabilitation, Ophthalmology, Genetics, Child and Maternal Health (DINOGMI), University of Genova, Genova, Italy; ^13^Child and Adolescent Neuropsychiatry Unit, Department of Medicine, Surgery and Dentistry, University of Salerno, Salerno, Italy; ^14^Department of Science of Health, School of Medicine, University Magna Graecia of Catanzaro, Catanzaro, Italy; ^15^Sleep Medicine Center, Neurological Mediterranean Institute IRCCS Neuromed, Pozzilli, Italy; ^16^Psychology Faculty, International Telematic University Uninettuno, Rome, Italy; ^17^Epilepsy Unit, Department of Human Neurosciences, Sapienza University of Rome, Rome, Italy

**Keywords:** AMPA receptors, AMPA antagonists, glutamate, perampanel, epilepsy, sleep, migraine

## Abstract

Glutamate is the brain’s main excitatory neurotransmitter. Glutamatergic neurons primarily compose basic neuronal networks, especially in the cortex. An imbalance of excitatory and inhibitory activities may result in epilepsy or other neurological and psychiatric conditions. Among glutamate receptors, AMPA receptors are the predominant mediator of glutamate-induced excitatory neurotransmission and dictate synaptic efficiency and plasticity by their numbers and/or properties. Therefore, they appear to be a major drug target for modulating several brain functions. Perampanel (PER) is a highly selective, noncompetitive AMPA antagonist approved in several countries worldwide for treating different types of seizures in various epileptic conditions. However, recent data show that PER can potentially address many other conditions within epilepsy and beyond. From this perspective, this review aims to examine the new preclinical and clinical studies—especially those produced from 2017 onwards—on AMPA antagonism and PER in conditions such as mesial temporal lobe epilepsy, idiopathic and genetic generalized epilepsy, brain tumor-related epilepsy, status epilepticus, rare epileptic syndromes, stroke, sleep, epilepsy-related migraine, cognitive impairment, autism, dementia, and other neurodegenerative diseases, as well as provide suggestions on future research agenda aimed at probing the possibility of treating these conditions with PER and/or other AMPA receptor antagonists.

## 1. Introduction

Epilepsy is a disorder characterized by spontaneous recurrent seizures, which may arise for different reasons (1). According to the most recent classification (2), epilepsy etiologies include structural, genetic, infectious, metabolic, immune, or even unknown causes. Each of these etiologies can initiate a series of different changes that disrupt the normal balance between excitation and inhibition (1). This imbalance, which could be caused by various possible alterations occurring within a neuronal network, is an established and well-accepted hypothesis for the pathogenesis of seizures and epilepsy (3). This hypothesis describes the major role of glutamate in transmitting an excitatory signal to other neurons (4). Approximately 70–80% of neurons are glutamatergic in the cerebral cortex. Cortical pyramidal neurons possess about 30,000 synapses, of which 95% are excitatory synapses. Thus, it is evident that glutamate is the main excitatory neurotransmitter in the brain and that glutamatergic neurons chiefly participate in fundamental neuronal networks. However, the pathological function of each glutamate receptor in epilepsy still has to be fully elucidated (4–8).

Among glutamate receptors, the ionotropic α-amino-3-hydroxy-5-methyl-4-isoxazole propionic acid receptor (AMPAR) is the predominant mediator of glutamate-induced excitatory neurotransmission in the central nervous system and, thus, appears as a major drug target for modulating several brain functions (1, 4, 6, 7). Indeed, their numbers and/or properties dictate the efficiency of glutamatergic transmission and underlie synaptic plasticity; AMPAR dysfunction is a major factor in many neurological and neurodegenerative diseases (1, 4, 7, 9).

α-amino-3-hydroxy-5-methyl-4-isoxazole propionic acid receptor antagonists have demonstrated antiseizure activity in various animal seizure models. However, to date, only perampanel (PER), a highly selective, noncompetitive AMPA glutamate receptor antagonist, has reached the market (10). In the United States, PER is approved as monotherapy and adjunctive therapy for focal onset seizures, with or without focal to bilateral tonic–clonic seizures, in patients aged ≥4 years, and for adjunctive treatment of generalized tonic–clonic (GTC) seizures in patients aged ≥12 years (11). In the EU, PER is approved for adjunctive treatment of focal onset seizures, with or without focal to bilateral tonic–clonic seizures, in patients aged ≥4 years and for GTC seizures in patients aged ≥7 years with idiopathic generalized epilepsy (IGE) (12).

However, preclinical and clinical data show how PER has the potential to address many other conditions, both within and beyond epilepsy (1). By following-up older hypotheses from our previous publication on the same topic (1), this article aims to review new preclinical and clinical data—particularly those produced starting from 2017—on PER, AMPAR-mediated synaptic transmission, epilepsy, and other neurological and neurodegenerative diseases, as well to provide suggestions on future research agenda aimed at probing the possibility of treating these conditions with PER and/or other future AMPAR antagonists.

## 2. PER so far: an overview of real-world evidence on focal and generalized seizures

The efficacy of PER in the treatment of epilepsy has been demonstrated in its registration trials. The first two real-world evidence studies, the “GENERAL” (13) and “FYDATA” (14), subsequently confirmed the effectiveness of PER in clinical practice for general and focal seizures, respectively. As these were the first studies, the enrolled population comprised patients with highly pharmaco-resistant epilepsy, and PER was being used in combination with ≥3 antiseizure medications (ASMs). Subsequent studies suggest that PER is even more effective when used as an earlier rather than late ASM (13–20).

Many other trials, observational studies, and meta-analyses have been conducted since PER approval in 2012; here, we will present a brief and not exhaustive overview of the studies addressing the treatment of focal and generalized seizures with PER.

### 2.1. Idiopathic and genetic generalized epilepsy

Idiopathic generalized epilepsies (IGEs) represent an important and common subgroup of epilepsies currently included in the wide chapter of genetic generalized epilepsies (GGE), whose definition and nosology have recently been revised by the ILAE *ad hoc* commission (21). IGEs include the following four syndromes: childhood absence epilepsy (CAE), juvenile absence epilepsy, juvenile myoclonic epilepsy (JME), and epilepsy with GTC seizures alone. Typical seizures encompass GTC, myoclonic, and absence (2, 22). These syndromes are often considered easy to treat with a good long-term prognosis for most patients (23), even if growing evidence has shown a variable rate of drug-resistant patients, especially in JME (24).

The approval of PER as adjunctive treatment of GTC seizures followed the positive outcomes of the randomized, double-blind, placebo-controlled phase III study 332 (25) by French et al. (26) enrolling patients with IGE and GTC seizures resistant to one to three ASMs. Patients were randomized to receive adjunctive PER once daily (up to 8 mg/day) or a placebo across 17 weeks (27). Patients who completed this first study could enter the second, open-label extension “OLEx phase” (27). During the double-blind phase, PER conferred a greater median percent reduction in primary GTC seizure frequency over 28 days and a 50% higher primary GTC seizure responder rate than placebo. During maintenance, 31% of PER-treated patients and 12% of patients receiving a placebo achieved primary GTC seizure freedom (26, 27). The magnitude of reduction in the frequency of myoclonic seizures was not higher than placebo, and the greater reduction in absence seizures in the PER group compared with placebo was not statistically significant (26, 27). These responses were maintained following the long-term (>104 weeks) adjunctive PER treatment of the OLEx phase (27). Similarly, 50 and 75% responder and seizure-freedom rates were maintained during long-term treatment (27). The efficacy outcomes reported during this long-term phase were generally consistent with real-world observational studies in patients with IGE treated with long-term PER (13, 26, 27).

Since both the Phase III 332 trial (25) and its extension phase (26) included few patients with myoclonic or absence seizures, the efficacy of PER on these seizure types could not be determined. Therefore, Villanueva and colleagues conducted a retrospective, multicenter, observational 1-year (GENERAL) study to analyze the tolerability and efficacy of PER across different seizure types in a large population with IGE (13). The 149 enrolled patients aged ≥12 years had a confirmed diagnosis of IGE and had been prescribed PER. The patient population included 60 with JME, 51 with GTC seizures only, 21 with juvenile absence epilepsy, 10 CAE, six adults with absence seizures, and one with Jeavons syndrome, with a mean age of 36 years. The PER most common dose was 4 mg/day (13). At 12 months, the retention rate was 83% (124/149), and the seizure-free rate was 59% for all seizures (88/149); 63% for GTC seizures (72/115), 65% for myoclonic seizures (31/48), and 51% for absence seizures (24/47). Compared with baseline, there was a reduction in seizure frequency at 12 months for GTC (78%), myoclonic (65%), and absence seizures (48%). PER was well tolerated, and seizure worsening was rare. The effectiveness of PER was good regardless of epilepsy syndrome, concomitant ASMs, and several prior ASMs (13). Actually, seizure freedom and retention rates were significantly higher when PER was used as an early add-on (after ≤2 prior ASMs) than late (≥3 prior ASMs). The authors concluded that the addition of PER in patients with IGE was associated with reductions in the frequency of GTC, myoclonic, and absence seizures, regardless of concomitant ASMs and epilepsy syndrome, and that PER should be used early to maximize the effectiveness in seizure freedom and retention rates (13).

Results regarding absence seizure are supported by another study that evaluated PER as the first add-on and second-line monotherapy in 20 subjects with CAE (16). Overall, 75% of patients were seizure-free with add-on therapy, and 60% remained seizure-free with PER monotherapy. Mild, transient side effects were reported only by two and did not lead to PER discontinuation. Moreover, PER did not negatively affect non-verbal intelligence, executive functions, emotional and behavioral symptoms, and parental stress (16).

In a study by Santamarina et al. (28), which enrolled 32 patients with IGE, the number of GTC, myoclonic, and absence seizures per month were significantly reduced by ≥87%, and there was a 75% reduction from baseline in the median number of generalized seizures per month, and 94 and 63% of patients were classified as responders and seizure-free, respectively.

Finally, preliminary data of a pooled analysis of 44 studies identifying 540 PER-treated IGE patients show responder and seizure freedom rates of 74 and 55%, respectively, and the proportions of patients with unchanged and worsening seizure frequency were 11 and 6%, respectively (29).

The PERMIT study, another pooled analysis of 44 real-world studies from 17 countries (30), has recently focused on the effectiveness, safety, and tolerability of PER in treating myoclonic seizures. This study included 156 patients with myoclonic seizures – 89% diagnosed with IGE, mostly (63%) JME—and showed a significant retention rate of 85%; responder rate and seizure freedom were 90 and 69% at 12 months and 86 and 63% at the last visit, respectively. Based on this evidence, the authors concluded that PER was an effective drug in treating myoclonic seizures in real-world experiences, particularly in IGE/GGE (30).

This observation supports PER use in this seizure type, especially in the context of drug-resistant JME, and expands the current therapeutic armamentarium for IGEs, which is quite limited compared with focal epilepsies, which might benefit from a much wider choice of ASMs. PER appears more effective when used as an early option, but randomized controlled clinical trials (RCTs) with larger samples are needed to confirm these results (Box 1).

BOX 1 **IGE and GGE: conclusions and future directions.**

- RCTs and real-world evidence studies showed that PER is effective in reducing GTC, myoclonic, and absence seizures.
- PER effectiveness is particularly higher when used as an early add-on.
- Future RCTs are needed to further clarify PER efficacy against absence seizures, as well as other generalized seizure types.
- Generally, PER should be included as an early therapeutic option in the limited armamentarium currently available for IGEs/GGEs.

### 2.2. Focal onset seizures

Several phase III RCTs and their open-label extensions demonstrated favorable efficacy and safety of PER in focal onset seizures (25, 31–36).

Regarding real-world evidence, the previously mentioned multicenter, retrospective, 1-year observational study FYDATA (14) enrolled patients with refractory focal epilepsies. In the efficacy population (*n* = 459), the median number of seizures significantly reduced at 12 months. For patients who completed the study (61%), seizure reduction was 100% for secondarily generalized seizures, 77% for simple partial seizures, and 58% for complex partial seizures. At 12 months, 40% of these patients were responders, and 10% were seizure-free (14).

In the study by Santamarina et al. (28), 113 patients with focal epilepsy were enrolled. At 12 months, there was a statistically significant reduction of ≥65% in the number of focal aware seizures, focal impaired awareness seizures, and focal to bilateral tonic–clonic seizures; a 65% decrease from baseline in the median number of focal seizures per month; and 82% of patients were responders, and 41% were seizure-free.

The PERADON study (18) enrolled 113 patients with focal onset seizures. Around a third received PER as a first add-on, while the others as a second add-on. At 12 months, 68 and 27% of the patients were responders and seizure-free, respectively, with high retention rates (>80%) and a significant reduction in the number of concomitant ASMs. The percentage of seizure-free patients at 12 months was significantly higher when PER was added as the first vs. second add-on.

In the PEREAGAL study (19), 77 patients with focal onset seizures were treated with PER for 12 months. 60% experienced a ≥ 50% reduction in seizure frequency, and 39% were seizure-free. Of the 20 patients with focal to bilateral tonic–clonic seizures, 12 (60%) achieved seizure freedom. Again, the responder rate was significantly higher when PER was given with one vs. two concomitant ASMs (72 and 45%, respectively).

In the PEROC study (20), all 503 enrolled patients (81% with focal epilepsy) received PER as the only add-on treatment to a background ASM. At 12 months, the median baseline seizure number normalized per 28 days of 1.84 was reduced to 0.07—a decrease of 99%; responders’ rate and retention rates were also high (84 and 89%, respectively); and almost half of the participants remained seizure-free. Once again, significant differences arose in patients using PER as an early add-on vs. late add-on, as they more often reached seizure freedom at the 3-month follow-up (66 vs. 53%). No major differences were observed in the other sub-analyses (Box 2).

BOX 2 **Focal onset seizures: conclusions and future directions.**

- RCTs and real-world evidence studies showed that PER is effective in focal onset seizures, especially in secondarily generalized seizures.
- Most observational studies suggest PER effectiveness to be particularly higher when used as an early add-on, as for IGE/GGE.
- Future RCTs are needed to further clarify PER efficacy against the different types of focal onset seizure.
- Generally, PER should be included as an early therapeutic option in the treatment of focal onset seizures to maximize clinical outcomes.

## 3. Mesial temporal lobe epilepsy with or without hippocampal sclerosis

Temporal lobe epilepsy (TLE) represents the most common form of focal epilepsy in adulthood (37). Based on ictal semiology, TLE is divided into two categories, the most common of which is mesial TLE (MTLE) (38). MTLE has traditionally been considered an acquired drug-resistant form of epilepsy, often associated with hippocampal sclerosis (HS), but a benign variant of MTLE has been recognized, usually easily controlled with a single ASM (38).

Data from animal models indicate a high potential effect of PER and AMPAR antagonists targeting the mesial temporal structures, mainly the hippocampal formation, due to enhanced expression of AMPARs in the reorganized epileptogenic hippocampus (1, 39). For example, kindling rat models of MTLE suggested an antiseizure effect of PER and confirmed its efficacy in inhibiting seizure initiation (40). Based on pre-clinical evidence, PER has been predicted to be particularly effective in MTLE patients, and more recently, some clinical studies support this hypothesis (1, 41).

### 3.1. Clinical studies on MTLE

The clinical effectiveness of PER in TLE patients in a real-life context has been reported for the first time in a multicenter cohort of 246 patients with focal epilepsy (77 with TLE, of whom 26 with HS) treated with PER as adjunctive therapy (42). Interestingly, the TLE group showed a greater seizure reduction at both timepoints of 6 and 12 months, and the presence of TLE predicted better outcomes in terms of seizure control (42). Another study evaluated the efficacy of PER in an MTLE homogeneous cohort of 37 patients (none with HS) as the first add-on option rather than a late add-on (15). Results showed that the former group had an 85% retention rate at 3 and 12 months, while the latter was 64% at 12 months. PER was particularly successful, especially when used as the first option in patients who failed the first ASM rather than after many ASMs (15). These results reinforce the idea of preferential PER efficacy in MTLE patients, even in monotherapy (15), and parallel those of Lin et al. (43). Lin et al. (43) described 44 MTLE patients (12 with HS) whose adjunctive PER treatment helped achieve clinically significant improvement, with a significant reduction in seizure frequency and a retention rate of ~73%. The global 50% responder rate was 47%, including complete seizure freedom in five patients (16%). No significant differences existed between groups of patients with and without HS (43). Recently, Nilo et al. (44) reported a greater efficacy of PER in focal lesional epilepsy rather than in non-lesional epilepsy, probably depending on the role of glutamatergic transmission in the pathological mechanisms of structural TLE.

Preclinical data and early clinical studies thus concur that PER is a valid (early) ASM option in patients with MTLE, even with HS. Further extensive studies on a larger number of patients with MTLE and a longer observation period are warranted (Box 3).

BOX 3 **MTLE: conclusions and future directions.**

- Data from animal models confirm a potentially high effect of PER targeting the mesial temporal structures, mainly the hippocampus.
- Few studies showed the preferential clinical efficacy of PER on MTLE patients.
- PER was significantly successful when it was used as the first option in patients who failed the first ASM rather than after many ASMs.
- Further extensive studies on a larger number of patients with MTLE and a longer observation period are warranted.
- *Post hoc* analysis considering the presence of HS across epileptic patients recruited in PER clinical studies is recommended.

## 4. Brain tumor-related epilepsy

It is estimated that about 20–40% of patients with a brain tumor have seizures, a condition known as brain tumor-related epilepsy (BTRE). Low-grade developmental brain tumors are those most commonly associated with epilepsy. Seizures are the onset symptoms in a significant proportion of cases, and this onset is relatively common in patients with brain metastases (45–47). Indeed, tumorigenesis and epileptogenesis share genetic, molecular, and cellular mechanisms representing “two sides of the same coin.” Such mechanisms include augmented neuronal excitatory transmission, impaired inhibitory transmission, genetic mutations in the *BRAF*, *IDH*, and *PIK3CA* genes, inflammation, hemodynamic impairments, and astrocyte dysfunction, which are still largely unknown (47).

Given this strict relationship, drugs able to target both seizures and tumors would be of extreme clinical usefulness. In this regard, ASMs are optimal candidates as they have well-characterized effects and safety profiles, do not increase the risk of developing cancer, and already offer well-defined seizure control (47).

### 4.1. The rationale for the use of PER

Irrespective of the causative origin, there is evidence supporting that the alteration of glutamate homeostasis plays a major role in both glial and glioneuronal tumor growth and tumor-related epileptogenesis mechanisms. Glutamate is produced in excess by tumor cells, enhancing their growth and survival. In this perspective, AMPARs play an important biologic role, as synaptic and electrical integration into neural circuits promotes glioma progression (48). Neuron and glioma interactions include electrochemical communication through *bona fide* AMPAR-dependent neuron–glioma synapses. Depolarization of glioma membranes promotes proliferation, whereas pharmacologically or genetically blocking electrochemical signaling inhibits glioma growth. Moreover, human intraoperative electrocorticography demonstrates increased cortical excitability in the glioma-infiltrated brain (49). These data suggest that AMPAR antagonists may not only reduce or abolish epileptiform activity in BTRE but also play a role in regulating glioma invasion and thus blocking this mechanism of tumoral growth, possibly in combination with systemic agents (48–51).

### 4.2. Preclinical data on tumor growth

In a well-designed experiment on glioma integration into a neural circuit, the use of PER resulted in an approximately 50% decrease in glioma proliferation in PER-treated mice compared with controls (48). In an *in vitro* study by Lange et al. (52), four ASMs with different mechanisms of action (levetiracetam, valproic acid, carbamazepine, and PER) were tested on patient-derived cell lines of glioblastoma and cell lines of brain metastases. Only PER showed systematic inhibitory effects on cell proliferation at rather low concentrations (10–30 μM), whereas all other drugs failed and reduced the high extracellular glutamate levels. Metastasis cells were much more resistant to treatment than glioblastoma cell lines. Glucose uptake was attenuated in all glioblastoma cells after PER exposure. However, cell death via apoptosis was not induced (52). In another *in vitro* study, Salmaggi et al. (53) investigated the effect of PER and temozolomide (i.e., a first-line chemotherapy agent for glioblastoma) in human glioma cell lines. Differently from Lange’s results, they found that treatment with 250 μM PER—or even with 100 μM in some cell lines—produced a marked increase in apoptosis. Another study found PER-induced apoptosis at concentrations as low as 10 and 1 μM in T98G and U-251MG cell lines. This experiment also showed a dose-dependent inhibitory effect of PER on cell viability and that the combination of PER and the SERPINE1 inhibitor tiplaxtinin (as tumor SERPINE1 overexpression may be related to poor prognosis) demonstrated further reduced cell viability in PER-resistant U-138MG cells, which have high expression levels of SERPINE1 (54). Moreover, both studies found a strong synergistic effect of the combination of PER with temozolomide (53, 54).

Differences in PER dosage, the analyzed cell lines, and apoptosis detection by different methods may partly account for some of these studies’ discrepancies. Such a pro-apoptotic effect is possibly due to the increased GluA2 and GluA3 expression after treatment with PER. In fact, modulation of AMPA receptor subunits has been described to modify the permeability of glioma cells to Ca^2+^, whereby the overexpression of calcium impermeable AMPA receptors subunit (i.e., GluA2) inhibited glioma cell motility and induced apoptosis (55).

Despite this evidence, in an *in vivo* study with a murine glioma model, PER was effective in abolishing tumor-associated epileptic events but did not affect tumor progression when combined with radiochemotherapy (56).

### 4.3. Clinical studies

So far, clinical studies of PER treatment in BTRE, summarized in Table 1, have focused only on its efficacy as an add-on therapy for glioma-associated seizures. Therefore, clinical data on the antineoplastic activity of PER are not available. Of these trials, the PERADET study is the largest and the only multicentric prospective study (62). A total of 36 patients were treated with PER as an add-on with a 12-month follow-up period. The study demonstrated statistically significant efficacy of PER at 12 months with a significant seizure reduction. At the end of 12 months, the responder rate (patients who experienced a ≥ 50% reduction in seizure frequency) of the 21 patients per-protocol population was 90%, with 33% being seizure-free. In the intention-to-treat group, the responder rate was 67% at the end of 12 months, with 25% of patients being seizure-free. PER was well tolerated (31% of patients experienced an AE, none was severe; three needed a treatment interruption). Regarding the response to the quality-of-life questionnaire for epilepsy (QOLIE 31-p), the questionnaire’s mean scores were in normal ranges at basal evaluation and remained stable at the final follow-up. PER in these patients maintained good efficacy over time, despite radiologically evidenced disease progression. Both patients with mutated *IDH1* and methylated *MGMT* appeared to respond better to PER treatment. This is in line with the study of Dunn-Pirio et al. (58), which found that most patients with decreased seizure activity had *IDH1*-mutant tumors. However, these results appear in contrast to an observational pilot study by Maschio et al. (63), as no significant differences were observed in the *IDH1*-mutated vs. wild-type and *MGMT* groups with or without promoter methylation. A pooled analysis of 44 clinical studies identified 127 BTRE patients (98% with focal-onset seizures) treated with PER. At 12 months, 71% were responders and 38% were seizure-free; only 12 and 3% had unchanged or worsening seizures, respectively (65). These results suggest that PER is effective when used to treat patients with BTRE (Box 4).

**Table 1:** Studies with perampanel add-on therapy for glioma-associated seizures.

Study	Enrolled patients	Perampanel therapy	Seizure response
Vecht et al. (57)	12 patients	2–12 mg/day	Seizure-free = 6/12
	Nine males, three females	Follow-up = 6 months	≥50% reduction = 12/12
	Median age = 41 years		Responder rate = 100%
Dunn-Pirio et al. (58)^†^	Eight patients	2–8 mg/day	Seizure-free = 0/8
	Six males, two females	Follow-up = 16 weeks	6/8 subjects observed benefits
	Median age = 45 years		Responder rate = N/A
Izumoto et al. (59)	12 patients	4–8 mg/day	Seizure-free = 6/10
	Six males, four females	Follow-up = 6 months	≥50% reduction = 10/10
	Median age = 59 years		Responder rate = 100%
Maschio et al. (60)	11 patients	7.3 mg/day	Seizure-free = 5/11
	Nine males, two females	Follow-up = 12 months	≥50% reduction = 9/11
	Median age = 54 years		Responder rate = 82%
Chonan et al. (61)	18 patients	2–4 mg/day for 17 patients	Seizure-free = 18/18
	Nine males, nine females	8 mg/day for one patient	≥50% reduction = 18/18
	Median age = 49 years	Follow-up = 10.6 months	Responder rate = 100%
Coppola et al. (62)	36 patients	2–12 mg/day	Seizure-free = 7/21
	23 males, 13 females	Follow-up = 12 months	≥50% reduction = 19/21
	Median age = 46 years		Responder rate = 90.4%
Maschio et al. (63)	26 patients	2–12 mg/day	Seizure-free = 7/21
	16 males, 10 females	Follow-up = 6 months	≥50% reduction = 20/21
	Median age = 47.5 years		Responder rate = 95.2%

† Baseline seizure frequency data were not collected. Only one participant completed the entire study, which was terminated early due to poor accrual.

Differences in the number of subjects in the Enrolled Patients and Seizure Response columns are due to patients dropping out of the study (64). Adapted from MDPI under the terms and conditions of the Creative Commons Attribution (CC BY) license.

BOX 4 **BTRE: conclusions and future directions.**

- Preliminary clinical data show how PER has an optimal antiseizure effectiveness profile in treating BTRE.
- The ability of PER to act on both mechanisms of epileptogenesis and tumor spreading and growth makes it particularly interesting in BTRE not only for seizure control but also as a possible combined antineoplastic strategy.
- Strong, well-designed preclinical studies with PER or other AMPAR antagonists are needed to demonstrate their potential double role in these two areas. If this hypothesis is confirmed, large, prospective clinical trials with effectiveness on seizure control, tumor response, and survival as primary endpoints are needed.

## 5. Status epilepticus

Status epilepticus (SE) is a major neurologic emergency that occurs in approximately 0.05–0.1% of the population. It is associated with significant mortality and morbidity, including neuronal death, cognitive dysfunction, and other systemic complications and consequences (66). The current treatment of SE involves benzodiazepines (e.g., diazepam, lorazepam) as a first-line treatment and phenytoin and fosphenytoin as a second-line treatment. Recently, the ESETT trial established that levetiracetam and valproate are also effective alternatives to phenytoin as second-line ASMs, demonstrating non-inferiority in stopping seizures after intravenous treatment (67). However, about 30–40% of SE episodes do not respond to administering first- and second-line treatments (68). Therefore, novel approaches targeting different mechanisms may help improve treatment outcomes (1, 69).

### 5.1. Preclinical data

The mechanisms underlying the transition from self-limited seizures to prolonged, medically refractory seizures are not fully understood. A loss of inhibitory GABA_A_ neuronal activity coupled with sustained glutamate-mediated excitatory activity due to alterations in NMDA and AMPA receptors is mainly known to be involved. In 2018, Leo et al. (70) extensively reviewed the role of AMPARs and their antagonists in SE in *in vitro* and *in vivo* models and human beings. They highlighted how different animal models showed relevant changes in the expression of AMPARs, particularly in their subunit composition, during the early stages of SE. Namely, early overexpression of calcium-permeable GluA2-lacking AMPARs has been found in excitatory synapses of principal cells of the piriform cortex and hippocampus in several animal models: the lithium pilocarpine (71), pilocarpine (72), and kainate-induced SE (73, 74), and kindling-induced seizures (75). Persistent expression of GluA2-lacking AMPARs on hippocampal pyramidal neurons during SE may also result in prolonged elevation of Ca^2+^ levels, which may contribute to excitotoxicity and potentially the development of epilepsy (74). Despite limited data on human beings, the review concluded that using AMPAR antagonists in patients with SE seems promising and that AMPAR antagonists, in particular PER, could become a new therapeutic option (70).

More recent preclinical data confirm and expand these previous findings. Beyond GluA2, the GluA1 subunit also appears to have an important role. The surface membrane expression of GluA1 subunit-containing AMPARs on hippocampal pyramidal neurons is increased in SE (76, 77), and AMPAR plasticity mediated by the GluA1 subunit plays a critical role in sustaining and amplifying seizure activity and contributing to mortality (77). As a result of molecular alteration in the machinery that regulates AMPAR function, synaptic plasticity is dysregulated, impairing long-term potentiation (LTP) in hippocampal synapsis and possibly leading to deficits in learning and memory that are frequently observed after SE in both animal models and human beings (78).

### 5.2. Clinical data

Despite the rationale to use PER in refractory and super-refractory SE, a systematic review published in 2018 highlighted how the response observed in the real-world clinical setting was only moderate (69). Several factors may have affected the efficacy results and partly explain the low response, including the small number and high clinical heterogeneity of the patients and the different dosages used. Furthermore, the lack of information on the type and etiology of SE and comorbidities prevented the exploration of the variables that may have influenced the efficacy of PER. In many studies, PER was given late in the course of SE; it was mostly used in patients with super-refractory SE, and the time from SE onset to the first PER administration ranged from 9.6 h to 35 days.

Furthermore, PER was administered after several attempts (up to nine) with other antiseizure or anesthetic drugs, suggesting that it was mostly used to treat SE associated with severe underlying brain dysfunction. The etiology of SE plays a crucial role in predicting SE outcomes (66) and is likely to be a major determinant of treatment response (79). When used to treat refractory and super-refractory SE, PER appears extremely well-tolerated, even at doses of up to 32 mg. No cardiorespiratory adverse effects were documented, and cholestasis (63%) (80) and asymptomatic increased liver enzymes (23% overall, 57% of patients receiving high doses) (81) were the only laboratory changes reported.

Since 2018, other clinical studies have been published, albeit none were RCTs. The type of the studies, patients’ characteristics, PER treatment characteristics, and PER response are summarized in Table 2. PER response ranged widely from 33 to 68%. This discrepancy between studies may depend on the criteria used to define treatment response, differences in enrolled patients, concomitant medications, the timing of PER addition, and other confounding factors. Out of the published clinical studies, the possible role of anti-glutamatergic drugs in stopping seizures in the context of post-anoxic SE deserves particular attention. The study by Beretta et al. (80) enrolled eight post-anoxic patients with super-refractory non-convulsive SE who were treated with PER (6–12 mg dose range). All patients had continuous electroencephalographic monitoring and favorable multimodal prognostic indicators. In six patients (75%), SE resolved within 72 h after administration of PER without changing the co-medication. More recently, similar findings in three patients with super-refractory SE after cardiac arrest were reported (87). These preliminary studies suggest a potential role in AMPA modulation of seizures after cerebral anoxia.

**Table 2:** Studies addressing the use of PER in status epilepticus.

Study	Type of study	Patients	PER treatment	SE resolution
Chen-Jul Ho et al. (82)	Retrospective, monocentric study	67 patients included; 22 received PER treatment (32.8%).	PER was given as a median 4th ASM (range 2–7) at a median of 5.45 days after SE onset (range 0.2–12.9 days).	8/22 (36.4%)
	Effectiveness criteria: Responder: seizure freedom (clinically or electrographically) within 4 days after PER administration (last ASM used), plus no relapse during hospitalization. NR: not seizure freedom within 4 days; PER was not the last ASM used; relapse during hospitalization.	Cerebrovascular diseases (8/22; 36%), head trauma (3/22; 14%), and autoimmune disorders (3/22; 14%) were the most common etiologies of SE.	Median initial dose: 2 mg (range 2–8 mg)—median maximum dose 4 mg (range 2–12 mg). Titration up to the maximum dose occurred in a median of 2 days.		More than half of the patients had a CSE (15/22; 68%).	No SAEs observed (four patients died of sepsis); no elevation in liver enzymes.
	Strzelczyk et al. (83)	Retrospective, multicentric study	1,319 patients included; 52 (3.9%) received PER treatment: one ESE (1.9%), 28 RSE (53.8%), and 23 SRSE (44.2%).				PER was given as a median 6th ASM (range 1–14) with a median latency from SE onset of 10 days (range 0.5–51 days).	19/52 (36.5%)
		PER response was defined as SE cessation for ≥24 h within 24 h from PER administration, without further administration of ASM or co-medications changes in the last 24 h.	Structural etiologies were the most common causes of SE (45/52; 86.5%); five patients with hypoxic SE (9.6%).		Median initial dose: 6 mg (range 2–24 mg)—median maximum dose 10 mg (range 4–24 mg): both significantly higher in NR patients.			
No SAEs were observed; two patients (3.8%) experienced dizziness and drowsiness.								
Alsherbini et al. (84)	Retrospective, monocentric, observational study	75 patients included and treated with PER: 31 DR (41.3%), 28 PR (37.3%), and 14 NR (18.6%).	PER was given as a median 6th ASM (range 5–7) with a median latency from SE onset of 1.8 days (range 0.9–3.3): no significant differences between DR and PR/NR.	31/75 (41.3%; median response time: 40 h [range 1–69])
	Effectiveness criteria: DR: the resolution of SE within 72 h of initiation of PER (with no additional ASMs). PR: the resolution of SE within 72 h of initiation of PER, but other ASMs were added to the treatment regimen within 12 h of PER initiation. NR: lack of resolution after PER initiation or multiple ASMs were added within 12–72 h following PER initiation.				Structural etiologies were the most common causes of SE (59/75; 78.7%).	Median initial dose: 12 mg (range 8–12 mg) – median maximum dose 12 mg (range 8–24 mg): no significant differences comparing DR and PR/NR.	More than half of the patients had an NCSE (52/75; 69.3%); DR had a higher percentage of patients with GCSE (41.9% vs. 22.7%; *p* = 0.08).	Seven patients (9.3%) experienced mild adverse effects: agitation (2), drowsiness (4), and liver enzyme elevation (2).
	Atsushiet al (85).	Prospective, monocentric, interventional study	22 patients included (mean age 59.5 years), all had a SE with the prominent motor phenomenon, either focal motor (8/22; 38%) or convulsive (14/22; 62%)		PER was administered after the failure of first- and second-line agents (or levetiracetam). NR patients received a third-line agent after PER.	15/22 (68%)		
		PER was considered effective in case of reduction in seizures frequency within 1 h from its administration (seizure free at 24 h).	Cerebrovascular diseases (8/22; 38%), CNS tumors (4/22; 18%) and head trauma (4/22; 18%) were the most frequent etiologies of SE		Oral loading dose: 8 mg.	
					No SAEs; only a few patients experienced mild adverse effects (drowsiness and dizziness).	
Slew-Na Lim et al. (86)	Retrospective, monocentric, observational	373 patients included; 81 received PER treatment (21.7%).	PER was given as a median 4th ASM (range 1–7); median latency from SE onset: 100 h (range 5–1,428).	27/81 (33.3%); (median response time: 40 h [range 1–69])
	Effectiveness criteria: Responder: seizure freedom (clinically/electrographically) within 72 h after PER administration and without other ASM or anesthetics changes. NR: ambiguous classification for co-medications changes.				Metabolic disturbances (27/81; 33.3%), cerebrovascular diseases (22/81; 22.7%), and CNS infections (12/81; 14.8%) were the most common etiologies of SE.	Median initial dose: 4 mg (range 2–36 mg)—median maximum dose 12 mg (range 4–36 mg). Initial and maximum dose was positively and negatively associated with response rate, respectively (OR 1.27, *p* = 0.025 vs. OR 0.74, *p* = 0.022).	More than half of the patients had a CSE (58/81; 71.6%).	No SAEs were observed even in case of high off-label dosage.

ARS, acute repetitive seizures; ASM, anti-seizure medication; CSE, convulsive status epilepticus; CNS, central nervous system; DR, definite responder; ESE, electrographic status epilepticus; GCSE, generalized convulsive status epilepticus; NCSE, non-convulsive status epilepticus; NR, non-responders; PER, perampanel; PR, partial responder; RSE, refractory status epilepticus; SAEs, serious adverse events; SE, status epilepticus; SRSE, super refractory status epilepticus.

Given the current results from preclinical and clinical studies, PER could be evaluated early as second-line therapy. However, PER is available only as an oral formulation, which can be administered via a nasogastric tube in patients with SE. Developing an injectable formulation would improve PER therapeutic possibilities, especially in the emergency setting. PER has a terminal half-life of approximately 105 h, allowing once-daily dosing and reaching a steady state in 10–19 days (88). Hence, if an initial dose of 6 mg is administered for the first time, PER will be on a therapeutic level only for a few hours (89), which might not be sufficient to control refractory or super-refractory SE in a short time and prevent seizure recurrence. These pharmacological considerations could represent the rationale for controlled testing for the efficacy and safety of PER given at dose intervals shorter than 24 h (89). Moreover, the arrival of the recently developed suspension formulation will probably soon lead to the publication of new, more accurate data that are still lacking (Box 5).

BOX 5 **SE: conclusion and future directions.**

- Preclinical and clinical evidence suggests PER as a therapeutic option in SE treatment, regardless of SE etiology or semeiology.
- PER was associated with a good safety profile even in high initial oral load cases.
- Lack of parenteral (e.g., intravenous) formulations limits the administration of PER, especially in the emergency setting.
- Further prospective studies with larger sample sizes, uniform designs, and common clinical protocols are needed to establish the role of PER in SE treatment.

## 6. Pediatric population and adolescents

In the EU, PER can be used in pediatric settings as an adjunctive treatment in patients aged ≥4 years with focal seizures, including those that evolve into bilateral tonic–clonic seizures. In the United States, it can be used as monotherapy. In the case of GTC seizures, PER can be used as add-on therapy in patients with IGE ≥7 years of age in the EU and ≥ 12 years in the United States (1).

Two meta-analyses of studies conducted in pediatric populations highlight the superiority of new ASMs over placebo as add-on treatments (90, 91). However, the authors also advocated for newer, better-designed RCTs using relevant outcomes, comparative designs, more reliable inclusion criteria, and appropriate follow-up length. Regarding studies using PER, only two (92, 93) could be included in these meta-analyses (90, 91).

The study by Rosenfeld et al. (93) is a pooled analysis of three core phase III studies (31, 32, 34) and their extension phase (35). Pooled data of 143 adolescents with drug-resistant partial seizures enrolled in these four studies showed that PER produced better seizure control and sustained short- and long-term seizure frequency improvements compared with placebo, especially when used at doses of 8 and 12 mg and concomitant with non-enzyme-inducing ASMs (93). The investigation by Lagae et al. (92) evaluated the effects of adjunctive PER compared with placebo on efficacy, safety, and behavior within study 235 (94). In this study, adolescents aged 12–17 years with partial-onset seizures and on a stable one to three ASM regimen were randomized to receive up to 12 mg/day of PER (85 patients on PER and 48 on placebo in total). The median reduction in seizure frequency from baseline was 58% for PER and 24% for placebo. PER treatment resulted in a 50% reduction in seizure frequency in 59% of patients compared to 37% of placebo. Changes in behavior as measured by the Child Behavior Checklist were minimal.

A retrospective, observational, multi-center study by Fernandes et al. (95) collected real-world data on the effectiveness and tolerability of PER throughout a 24-month follow-up period in patients with epilepsy. Subgroup analyses considering pediatric patients (<18 years; *n* = 26) and adult patients (*n* = 68) initially suggested a significant difference in seizure reduction at the 24-month follow-up visit. However, after *post hoc* analysis corrections, the difference became not significant. Therefore, the authors concluded that PER treatment does not appear to be distinguished by significant differences between adult and pediatric patients. These data also suggest a trend in higher efficacy of PER among younger patients, but further studies are warranted (95). A more recent study is the interim analysis of 73 preadolescents (1 to <12 years) and 97 adolescents (12 to <18 years) enrolled in the PROVE study (96), a retrospective, phase IV study assessing dosing, efficacy, retention, and safety of PER administered in routine clinical care (97, 98). After 2 years of PER treatment, 43% of preadolescent patients continued PER, and the median seizure frequency percentage was reduced by 98%. Over the entire 2-year period, >38 and > 34% of preadolescent patients experienced a ≥ 50% and ≥ 75% reduction in seizure frequency, respectively. Moreover, freedom from a seizure was achieved by 27, 33, and 25% of patients at the end of 12-, 18-, and 24-month treatment, respectively (98). Regarding the adolescent patients, 56% remained on PER at 24 months, and the median seizure frequency percentage was reduced by 80%. Over the entire 2-year period, >38 and > 30% of adolescent patients experienced a ≥ 50% and ≥ 75% reduction in seizure frequency, respectively. Moreover, freedom from seizures was achieved by 22, 18, and 33% of patients at the end of 12-, 18-, and 24-month treatment, respectively (98). A sub-analysis of the PERMIT study identified 64 pediatric and 204 adolescent patients treated with PER. At the last visit, seizure freedom and responder rates were 24 and 54%, respectively, in pediatric patients, and 23 and 56%, respectively, in adolescents (99). Another retrospective study comprised 133 patients with a median age of 15 years. 15% achieved seizure freedom, and 39% gained ≥ 50% seizure reduction. The relapse-free survival in responders was 69% at 12 months and 30% at 36 months. The presence of epileptic encephalopathy and cognitive impairment was significantly associated with PER failure.

Overall, data from these studies indicate that PER is efficacious and well-tolerated in the pediatric setting, especially in partial-onset seizures and in adolescents, and outcomes in this younger population are comparable to those in the whole population. The presence of epileptic encephalopathy and cognitive impairment should be kept in mind, particularly at younger ages, to optimize the use of PER in the clinical setting (92, 93, 98, 100).

### 6.1. The effects of PER on cognition in the pediatric population

Impairments in cognitive domains are common in epilepsy (101). It is particularly important to evaluate the cognitive effects of ASMs in children and adolescents, as cognitive and executive functions contribute to a good adaptation to social and school life and a good quality of life (QoL) (102). Visuospatial skills, also known to be impaired in patients with epilepsy, are pivotal in several contexts, such as movement, spatial orientation and representation, non-verbal communication, geometric recognition, graphic production, and various problem-solving and learning achievements (103).

Table 3 provides an overview of the type of study, patients, treatment regimen, and cognitive effects of all available studies that used standardized scales to evaluate the effect of PER on pediatric cognition. The currently available data seem encouraging, as it appears that PER has no negative effects on the global cognitive profile (94) and executive functions, such as working memory and attention (108). In a study using quantitative electroencephalogram (qEEG, an established technique used to evaluate the effect of ASMs on EEG background activity and cognition), a significant increase in beta1 and total beta bands was found in children (and adults as well), suggesting a beneficial effect of this drug on cognition and alertness, although the sample size was small (110, 111). Regarding visuospatial abilities, Operto et al. published the first study addressing their changes through standardized tests during PER therapy (103). In the 42 treated patients assessed at baseline and after 12 months, mean scores on the Rey–Osterrieth Complex Figure Test remained almost unchanged for both visuospatial memory and perception skills, suggesting that PER did not impair these functions. This finding is important because visuospatial perception and memory were significantly correlated with non-verbal intelligence and executive functions. Moreover, they found that visuospatial abilities were not significantly related to age, sex, age at onset of epilepsy, seizure frequency, epilepsy duration, side and lobe of seizure onset, and ASM number (103). A review addressing the neurocognitive effects of various ASMs in children and adolescents with epilepsy found that, overall, PER treatment did not appear to be associated with broad cognitive deficits in pediatric patients, and the attentional deficits with increasing exposure to PER reported in one placebo-controlled trial (94) were found to be associated with other clinical factors, including seizure variables and concomitant medication (102).

**Table 3:** Studies using standardized scales to evaluate the effect of PER on cognition in pediatric populations.

Study	Type of study	Patients	Treatment	Cognitive effects	Authors’ conclusions
Meador et al. (94)	Randomized, double-blind, placebo-controlled, parallel-group phase II study	133 adolescents (12 to <18 years) with an IQ ≥70 and a diagnosis of partial-onset seizures	79 patients treated with PER, 44 with placebo	CDR System Global Cognition Score (overall *p* > 0.05):	Favorable cognitive profile for PER
				Power of attention (*p* > 0.05)	
	19-week follow-up				
				Working memory (*p* > 0.05)	Additional studies are needed to compare PER with other ASMs
			PER: 8–12 mg/day	Quality of episodic memory ↑ (*p* = 0.012)	
				Continuity of attention ↓ (*p* = 0.013)	
				Speed of memory ↓ (*p* = 0.032)	
			1–2 other ASMs	Letter fluency (*p* > 0.05)	
				Category fluency (*p* > 0.05)	
				LGPT (*p* > 0.05)	
Piña-Garza et al. (104)	Open-label extension phase of the trial by Meador et al. (94)	Patients who completed all scheduled visits in the double-blind phase in the study by Meador et al. (94) were eligible	Same as Meador et al. (94); those assigned to placebo switched to PER 2 mg/day, which was up-titrated weekly in 2-mg increments, up to a maximum of 12 mg/day	CDR system global cognition score (overall *p* > 0.05):	PER did not significantly affect cognitive parameters, except for the power of attention. No clinically meaningful effects on growth and development
				Power of attention ↓ (*p* = 0.03)	
	The extension phase comprised Part A (a 6-week double-blind conversion period and a 27-week open-label maintenance period) and Part B (additional open-label extension of 15–52 weeks)				
				Working memory (*p* > 0.05)	
				Quality of episodic memory (*p* > 0.05)	
				Continuity of attention (*p* > 0.05)	
				Speed of memory (*p* > 0.05)	
				Letter fluency (*p* > 0.05)	
				Category fluency (*p* > 0.05)	
				Lafayette Grooved Pegboard test (*p* > 0.05)	
Villanueva et al. (105)	Report aiming at characterizing the PK profile of PER and the relationship between PER plasma concentration and cognitive function	110 adolescents with the same characteristics as in Meador et al. (94)	Same as in Meador et al. (94)	CDR system global cognition score (overall *p* > 0.05):	No significant relationship between PER exposure and overall cognitive function
				Power of attention (*p* > 0.05)	
				Working memory (*p* > 0.05)	
				Quality of episodic memory ↑ (*p* < 0.05)	
				Continuity of attention ↓ (*p* < 0.05)	
				Speed of memory (*p* > 0.05)	
Fogarasi et al. (106)	Global, multicenter, open-label, single-arm study	180 patients from 4 to 12 years of age	Mean PER dose 7.0 mg/day (range 2–16 mg/day)	No clinically significant changes at week 23 from baseline as assessed by ABNAS in total score and each of the domains	PER did not produce any clinically significant changes in cognitive function at week 23 compared with baseline
	To evaluate the effects of PER on cognitive function, secondary safety endpoints included changes from baseline in ABNAS at week 23		PER used as oral suspension (0.5 mg/mL)		
Majid et al. (107)	Report aiming at exploring the PER exposure–response relationships for cognition and safety with data from (Fogarasi 2020)	156 PER-treated subjects aged 4 to <12 years with partial-onset seizures or primary generalized tonic–clonic seizures	Same as in Fogarasi et al. (106)	No discernible relationship between PER and change from baseline for ABNAS, CBCL, or LGPT	Cognitive function is not clinically impaired by PER administration
Operto et al. (108)	Observational single-center study	37 patients aged 12–18 years with focal pharmacoresistant epilepsy already in therapy with 2 or 3 ASMs	PER was added with 1 mg/week increments up to a dose of 2–4 mg/day	EpiTrack Junior test: no significant differences from baseline at 6 (*p* = 0.137) and 12 months (*p* = 0.051), although there was a trend for improvement at 12 months	PER therapy did not significantly influence attention and executive functions; on the contrary, it was possible to highlight a slight improvement in cognitive performance. Even the emotional and behavioral profile has not changed after PER, and no significant adverse effects on behavior have been reported
	12-month follow-up			7/30 patients had achieved better scores on the EpiTrack Junior after 12 months, and 22/30 had no significant changes	
				Significant improvements in executive function occurred more frequently than worsening at 6 months (13 vs. 6%) and 12 months (23 vs. 3%)	
Operto et al. (108)	Prospective observational study	46 adolescents aged 12–18 years with focal and generalized drug-resistant epilepsy already in therapy with 1–2 ASMs	PER dose: 2–8 mg/day (mean dose = 3.40 ± 1.17)	No changes in the Rey-Osterrieth Complex Figure Test at 12 months compared with baseline	Visuospatial memory and perception were not significantly affected by PER therapy. These results suggest that PER has good tolerability in adolescence, even in the medium/long term
	12-month follow-up				
Auvin et al. (109)	Prospective cohort study	13 patients with Lennox–Gastaut Syndrome. Mean age at the start of PER treatment: 12.8 years (median, 13; range, 6–18.5). Patients had received prior treatment with 6–9 different ASMs	PER was initiated at 2 mg/day and titrated to a median maximum dose of 6 mg/day (range, 4–8)	Parents and physicians reported improvements in cognitive function and/or behavior for seven patients (53.8%) during the physical examination as part of follow-up, parallel to reductions in seizure frequency	No formal conclusions on cognitive function and behavior, as they were not formally assessed but relied on anecdotal observations
	Mean follow-up duration: 10.8 months (range, 1–24 months)				

↑, increase. ↓, decrease. ABNAS, A-B neuropsychological assessment; ASMs, antiseizure medications; CBCL, child behavior checklist; CDR, cognitive drug research; IQ, intelligence quotient; PER, perampanel.

Partly drafted with data from (102).

In conclusion, beyond its efficacy in reducing seizures in children and adolescents, PER also appears not to hinder the cognitive and executive domains. Therefore, it can also be considered a safe and valid treatment in this population from the perspective of cognitive abilities (102, 103) (Box 6).

BOX 6 **Pediatric population and adolescent: conclusions and future directions.**

- PER has shown efficacy in pediatric settings, especially in adolescents, and in treating partial-onset seizures, with outcomes comparable to the older population.
- Data from RCTs in pediatric settings are scarce, especially those involving very young patients (age < 12 years).
- Evidence from the literature suggests that PER does not cause significant changes in the cognitive profile, executive functions, and visuospatial skills of children and adolescents with epilepsy.
- Studies evaluating cognitive function in pediatric patients taking PER alone would be useful.
- Neuroimaging studies are lacking and needed to supplement neuropsychological data.

## 7. Elderly population

Epilepsy has a peak incidence in older age groups, with an annual incidence of 134 per 100,000 in people aged ≥65 years (112, 113). Due to the rapidly aging population, epilepsy in the elderly is increasingly encountered in clinical practice. Elderly patients are, however, underrepresented in regulatory epilepsy trials due to comorbidities, difficulties in recruitment, and problems in providing informed consent. Issues can also be related to the causes of seizures, as about 25% of older people who develop epilepsy have no defined etiology, resulting in the diagnosis of “late-onset epilepsy of unknown etiology” (113). Accordingly, real-world studies are needed to collect additional data on the efficacy and safety of ASMs in this population (113). Age-related changes can influence ASM pharmacokinetics, pharmacodynamics, and side effects. Some ASMs can induce or inhibit hepatic cytochrome P450 enzymes resulting in detrimental metabolic effects or drug–drug interactions, which are major concerns in elderly patients as they often have multiple diseases with multiple drug therapies (113). Thus, the newest ASMs could represent promising options for treating epilepsy in the elderly. Of note, PER has a favorable pharmacokinetic profile with a low potential for drug–drug interactions and can improve treatment adherence due to a once-daily formulation (113).

### 7.1. Clinical data

Few clinical data are available on using PER in elderly patients with epilepsy (Table 4). Pooled analyses of five interventional phase III/IV studies in which PER was used as adjunctive (studies 307 and 335), first add-on (study 412), or monotherapy (study 342) included 109 elderly patients (age ≥ 60 years) treated with PER (17, 117). The efficacy of PER was consistent with that observed in the adult population. AE rates were similar between elderly and adult patients (85 vs. 77%). However, as risks of falls, dizziness, and fatigue were greater in the elderly, careful titration of PER was suggested, especially at higher doses considering the dose-related risk of falls (118).

**Table 4:** Clinical outcomes and adverse events regarding the use of PER in elderly patients with epilepsy.

Study	Type of Study	Population aged ≥65 years	Seizure freedom	Seizure response (perampanel daily dose)	Retention rate	Adverse events	Common adverse events
Leppik et al. (100)	Pooled analysis of three phase III trials	*n* = 20	n/a	22.2% (12 mg); 42.9% (8 mg); 0% (4 mg); and 100% (2 mg)	NA	85%	Dizziness (45%), fatigue (25%), and falls (25%)
Rohracher et al. (114)	Pooled individual real-world studies	*n* = 135 (*n* = 46 for seizure freedom)	28.3%	n/a	47.8%	79.4%	Dizziness (24.7%), behavioral psychiatric (16.5%), somnolence (16.5%), falls/ataxia (8.2%), and fatigue (8.2%)
Rohracher et al. (115)	Prospective audit	*n* = 20	35%	n/a	75%	35%	Fatigue 20%, vertigo (15%)
Lattanzi et al. (116)	Retrospective, Italian multicenter study	*n* = 92	23.9%	57.6% (4 mg at 3 months; 5 mg at 6 months; and 6 mg at 12 months)	78.3%	34.8%	Irritability (8.7%), somnolence (4.3%), and dizziness/vertigo (4.3%)

NA, not available.

The multicenter study by Lattanzi et al. (116) is, so far, the most extensive real-world study on the elderly. Consecutive patients who were prescribed PER therapy were enrolled. The primary objective was to assess the 12-month effectiveness of adjunctive PER in patients older than 65 years. Among the 92 elderly patients, the most commonly prescribed daily doses of PER at 12 months were 4 and 6 mg. 24% of patients had a reduction in the dosage of one or more concomitant ASMs, and withdrawal of one or more concomitant ASMs occurred in 33%. Seizure frequency reduction at a baseline of at least 50% and seizure freedom were achieved by 58 and 24% of patients, respectively. PER withdrawal occurred in 22% of patients, mostly due to AEs; of these, 50% had their treatment discontinued at 3 months, 15% at 6 months, and 35% at 12 months (Table 4). The rate of patients experiencing behavioral and psychiatric AEs was significantly higher in patients with psychiatric comorbidities (23 vs. 8%). There were no differences in behavioral and psychiatric AEs according to the concomitant use of levetiracetam and the history of cognitive decline, either mild cognitive impairment or dementia (116).

The study by Rohracher et al. (81) is a pooled, individual-level analysis of observational studies of PER in routine clinical practice in 45 specialized centers. The decision to prescribe PER was made by the treating physician based on clinical need and suitability, while individual centers and investigators set their own inclusion criteria. PER starting dose was 2 mg, and the median dose at 12 months was 6 mg. At 12 months, 48% of participants remained on PER. Discontinuation was due to intolerability in 24% of patients, lack of efficacy in 6%, both intolerability and lack of effectiveness in 3%, other reasons in 3%, and unspecified in 16%. At 12 months, 28% of patients with evaluable data were seizure-free for at least 6 months. The seizure-free rate was 10%. AEs were reported by 79% of patients with evaluable data. The percentage of patients reporting one or more AE was 24% for psychiatric AE, 33% for cognitive AE, 34% for somatic AE, and 3% forAEs related to weight/appetite change (114).

A prospective audit by Rohracher et al. (115) evaluated the efficacy and tolerability of PER in 20 elderly patients. Compared with 65 younger patients, older patients at baseline had a lower PER dosage (2–8 mg vs. 2–12 mg), lower concomitant ASMs, and lower monthly seizure frequency. In the 57 months of follow-up, they showed significantly higher seizure freedom (35% [7/20] vs. 13.8% [9/65]), and, albeit non-significant, higher retention rate (75% [15/20] vs. 53.8% [35/65]) and lower AEs (35% [fatigue 20%, vertigo 15%] vs. 55.4% [vertigo 40%, psychiatric effects 9.2%]) (115, 119).

A study by Liguori et al. (116) analyzed a subgroup of 10 patients aged ≥60 years in whom PER was used as a first or second add-on. After 12 months of follow-up, four patients (40%) were seizure-free, two (20%) achieved ≥50% reduction in seizure frequency from baseline, two (20%) discontinued treatment due to AEs, and one (10%) due to ineffectiveness.

Finally, in the FYDATA study (14), logistic regression revealed that patients aged ≥65 showed a better clinical response to PER than younger patients (Box 7).

BOX 7 **Elderly: conclusions and future directions.**

- PER was effective and well-tolerated in elderly patients with predominantly drug-resistant, focal epilepsy treated in a real-world clinical setting.
- No unexpected AEs occurred throughout the real-world follow-up.
- Slow titration rates are advisable.
- A response may occur at low doses (reassess seizure control when 4 mg daily dose is reached before any increase if required).
- Real-world studies are needed to formally assess PER effects on cognitive functioning.

### 7.2. Neurodegeneration and epilepsy

There is a deep intertwining between seizures and cognitive decline. People with epilepsy have a 3-fold increased risk of dementia compared with the general population, even higher in the case of late-onset epilepsy (112). Amyloid-beta (Aβ) 1–40 and 1–42 peptides, the main components of senile plaques, are major actors in the pathophysiology of several neurodegenerative diseases, such as Alzheimer’s disease. Moreover, Aβ has been shown to promote seizures in experimental studies because it possesses pro-epileptogenic activity already at the oligomer stage (112).

#### 7.2.1. Preclinical data

Neurons’ distance from Aβ plaques affects the proportion of silent, normal, and hyperactive neurons (120). In fact, Aβ regulates glutamatergic currents mediated by NMDARs and AMPARs (but without affecting GABA currents), inhibits synaptic currents, and disrupts synaptic plasticity (121–124). Aβ is known to reduce surface AMPAR expression (124–126), especially with regard to the GluA1 subunit (127), as well as to induce and enhance AMPAR internalization, ubiquitination, and degradation (128). Glutamate clearance rates are reduced in synapses close to amyloid deposits, and chronic states of elevated glutamate levels are found near amyloid plaques (129). It has been shown that plaques and Aβ oligomers cause aberrant excitatory neuronal activity, epileptiform activity, and seizures at the network level, despite reducing synaptic currents and AMPAR-postsynaptic expression (112, 130–132). Cellular mechanisms of these contradictory Aβ-dependent effects have not been completely elucidated (112, 133). Neural activity can be enhanced by repressing inhibitory synapses onto excitatory neurons even if glutamatergic synapses on excitatory cells are depressed. Another hypothesis is that hyperexcitability can be driven by the Aβ-induced suppression of glutamate reuptake (126). In any case, *in vivo* studies have demonstrated the pro-epileptogenic properties of Aβ oligomers, especially in hippocampal neurons (134, 135). A single intracisternal Aβ injection is sufficient to facilitate seizures (136), and Aβ dimers can increase firing in hippocampal CA1 neurons (137). In another murine model (138), changes in extrinsic and intrinsic neuronal properties and dentate gyrus transmission occurred under the age of 3 months, when plaque deposition has not yet begun, as well as lowered hippocampal seizure threshold, impaired hippocampal LTP, and decreased hippocampal dendritic spine density (127, 139–141). Eventually, Aβ accumulation might result in more neurons adopting a more active phenotype. This epileptiform activity will, in turn, facilitate Aβ deposition in hippocampal neurons, triggering an Aβ-driven vicious circle (112, 123, 142).

According to this view, strategies able to prevent both Aβ-induced epileptogenic changes and seizure-induced Aβ alterations are of extreme interest, as they would allow tackling not only epileptogenesis but also neurodegeneration. From this perspective, ASMs have been widely investigated because some of them not only reduce cortical hyperexcitability but might also directly affect the Aβ cascade, including plaque deposition (112). Preliminary data of PER show interesting results from this perspective. The study by Bellingacci et al. (143) investigated whether the modulation of AMPARs counteracted the alteration of hippocampal epileptic threshold and synaptic plasticity linked to Aβ oligomers accumulation by using both an *in vitro* model of epileptic-like activity and an *in vivo* model of amyloidosis. *In vitro*, Aβ-induced hyperexcitability was counteracted by low PER doses, which, *per se*, did not affect physiological synaptic transmission. In parallel, the reduced *in vivo* epileptic threshold found in Aβ oligomers-injected mice was restored by PER-induced mild modulation of AMPARs. PER also restored Aβ-induced impairment of hippocampal LTP *in vitro* and significantly improved hippocampal-based cognitive performances of Aβ-lesioned mice. These findings suggest PER’s usefulness in reducing hippocampal networks’ hyperexcitability and synaptic plasticity deficits induced by Aβ oligomers accumulation (143).

#### 7.2.2. Clinical data

Late-onset epilepsy of unknown etiology, epileptic prodromal AD, and seizures in AD are all possible clinical correlates of an Aβ-driven continuum, spanning epilepsy and cognitive decline (112). Indeed, late-onset epilepsy, often accompanied by Aβ pathology, carries a high risk for dementia, and seizures frequently occur in people with prodromal AD (112). Unfortunately, no clinical trial with PER has been conducted so far, and it seems that none is being planned. Observational studies are also missing. Therefore, clinical data are lacking on using PER to tackle dual neurodegeneration epilepsy. Recently, a peculiar case report has been published (144). An 89-year-old woman with severe AD dementia, psychiatric symptoms, and intractable myoclonic epilepsy refractory to different ASMs, was given PER. Shortly after, myoclonus and psychiatric symptoms improved without adverse effects. The authors concluded that PER might be useful for controlling intractable epilepsy accompanied by AD, but rigorous, well-designed studies with a sufficient number of patients are necessary (144) (Box 8).

BOX 8 **Neurodegeneration: conclusions and future directions.**

- Seizures and dementia-related cognitive decline are deeply intertwined. Indeed, people with epilepsy have a higher risk for dementia and vice versa.
- Preclinical data support this relationship, as Aβ facilitates epileptiform activity, and epileptiform activity facilitates Aβ deposition, resulting in a vicious cycle.
- ASMs might be useful in tackling this vicious circle. In a preclinical study, PER reduced hippocampal networks’ hyperexcitability and synaptic plasticity deficits induced by Aβ oligomers accumulation.
- Preclinical and clinical data on using PER in this setting are scarce or lacking, respectively.

## 8. Rare diseases

Perampanel might be used to treat some rare genetic epilepsies, such as those with loss of GABA inhibition (e.g., SCN1a), overactivity of excitatory neurons (e.g., SCN2a, SCN8a, and KCNQ2), and variants in glutamate receptors (e.g., GRIN2a). Data from a retrospective study comprising 137 patients with 79 different epilepsy etiologies are available (145). The mean reduction in seizure frequency was 57% ± 34%. 44% of patients sustained >75% reduction in seizure frequency, including 28% with >90% reduction in seizure frequency (particularly those with GNAO1 and PIGA etiologies). The etiologies showing the highest PER efficacy included SCN1A, GNAO1, PIGA, PCDH19, SYNGAP1, POLG1, POLG2, and NEU1, probably due to a targeted effect related to glutamate transmission (146).

### 8.1. Progressive myoclonus epilepsy

Progressive myoclonus epilepsies (PMEs) comprise different genetically heterogeneous diseases, such as Unverricht-Lundborg disease (EPM1, MIM #254800), Lafora disease (EPM2, #254780), sialidoses (#256550), and other rare disorders (147). The most frequent symptom is stimulus-sensitive multifocal cortical myoclonus, mainly occurring during active movements. Other frequent symptoms include myoclonic and tonic–clonic seizures, possible cognitive decay, and variable ataxia (148).

The biggest interventional study on PER in PMEs was by Canafoglia et al. (148), enrolling 49 patients with PME of various etiologies. The severity of myoclonus assessed before and after 4–6 months of the steady PER dose was reduced. Convulsive seizures that recurred at least monthly were reduced by >50% in 17 patients. PER was more likely to improve outcomes in patients with EPM1 or EPM1-like phenotype (148).

Recently, Assenza et al. (149) published a case series and meta-analysis on studies using PER in PMEs. Beyond the study by Canafoglia et al., the largest study included, they found 10 longitudinal retrospective case–control series (148). Most patients reported a significant improvement of action myoclonus and subsequently improved independence after PER treatment, representing a valid adjunctive ASM for various forms of PME. This would expand the restricted armamentarium available for these conditions, for which the commonly used sodium channel blockers are not recommended (149).

### 8.2. Lennox–Gastaut syndrome

The rare condition known as Lennox–Gastaut syndrome (LGS) is a severe developmental and epileptic encephalopathy with childhood-onset and is associated with high morbidity and detrimental effects on the QoL of patients and families (145, 150). LGS, in which seizure freedom might be unachievable, is mostly treated with polytherapy, and side effects often affect the QoL more than seizures themselves (151). Although valproate is still the preferred first-line choice, often combined with clobazam or lamotrigine, PER is usually within the off-label treatments (145, 152). PER has been investigated as adjunctive therapy in patients with inadequately controlled seizures associated with LGS (153, 154), but the sponsor terminated the study early due to recruitment challenges, further impacted by COVID-19. To date, preliminary results from one clinical trial only are available. The median percent reduction in drop seizure frequency/28 days from baseline for PER vs. placebo was 54 vs. 8% and 27 vs. 5% for total seizures. The 50% responder rate for drop seizures for PER vs. placebo was 56 vs. 32%, and 38 vs. 26% for total seizures (155). The efficacy and safety of PER in LGS patients are suggested by outcomes from retrospective, open-label, observational studies (109, 156–161). The largest and most recent study specific to LGS included 87 patients who received adjunctive PER. 41% of the whole patient cohort were responders, of whom 61% experienced a ≥ 50% reduction in the frequency of drop attacks (median follow-up of 11 months), and 36% experienced seizure relapse over 36 months. The probability of remaining responders was 89% at 3 months and dropped to 62% at 36 months, suggesting a possible partial loss of efficacy in a few patients over time. At the end of the follow-up, 26% of patients of the whole cohort were responders and did not have seizure relapse. Importantly, add-on PER allowed the discontinuation and/or dosage reduction of other treatments in 37% of the patients (161).

Two other specific studies on LGS reported response rates (≥50% seizure reduction) of 69% in children or adolescents (109) and 65% in adult patients (157). Moreover, behavioral and/or cognitive function improvements were reported in 54% of children and adolescents (109) and 6% of adult patients (156). In another study, one patient with LGS was classified as a partial responder after 24 months of adjunctive PER treatment (158). Finally, one study on developmental and epileptic encephalopathies, which mostly included patients with LGS or Lennox-like syndrome, found that PER was effective in reducing seizures, especially in GTC, tonic, and focal-onset seizures, as well as in seizure clusters (160).

Thus, real-world evidence suggests that PER is efficacious and generally well tolerated as an adjunctive treatment for LGS-associated seizures. However, randomized, controlled trials are needed to validate these findings (109, 152, 156, 161).

### 8.3. *SCN1A* spectrum and Dravet syndrome

Dravet syndrome is a genetic developmental epileptic encephalopathy caused mainly by mutations in the *SCN1A* gene (162).

#### 8.3.1. Preclinical data

One study evaluated the effect of PER on a mouse model of Dravet syndrome (*SCN1A* E1099X/+). Treatment with PER 2 mg/kg attenuated epileptic activity and inhibited spontaneous recurrent seizures. PER significantly ameliorated seizure frequency and discharge duration and increased temperature tolerance in a hyperthermia-induced seizure experiment. A cross-over study was also carried out, demonstrating that the decreased susceptibility and severity of the hyperthermia-induced seizures were due to the PER therapy and were not caused by individual differences (163).

#### 8.3.2. Clinical data

To the best of our knowledge, very few patients with *SCN1A* spectrum/Dravet syndrome treated with PER are reported in the literature. One retrospective study included 10 patients taking two to four concomitant ASMs. Seizure frequency was reduced by over half in 50% of the patients. PER effects occurred between 3 and 6 months following the treatment initiation. Seizure reduction was observed at 0.1 ± 0.07 mg/kg/day (164). In the study by Nissenkorn et al. (146), 11 of 17 (65%) patients with Dravet syndrome due to an SCN1A pathogenic variant were responders, and 35% of them had >90% seizure reduction. In another retrospective study enrolling three patients with Dravet syndrome, two had a 50% seizure reduction (165). Another patient was a 12-year-old girl who achieved complete resolution of her spontaneous seizures over 5 years after starting PER, despite previous use of different ASM combinations (166). Lastly, another female patient developed frequent myoclonic and apneic ASM-refractory seizures during the neonatal period due to an *SCN1A* mutation-induced encephalopathy and had to undergo a tracheotomy. However, when PER was added, apneic seizures ceased (167).

## 9. Sleep

### 9.1. Sleep function, learning, and AMPA receptors

Sleep and AMPARs play a major role in learning and synaptic homeostasis through synaptic downscaling (168, 169). AMPAR synaptic expression is 30–40% higher after wakefulness than after sleep in rats, and phosphorylation changes of AMPARs are also consistent with net synaptic potentiation during wake and depression during sleep (170). Glutamate levels also change as a function of the behavioral state. Specifically, glutamate concentration increases progressively during waking and rapid eye movement (REM) sleep. It decreases progressively during non-REM (NREM), indicating long-term homeostasis of extracellular glutamate across sleep-waking states (171).

According to these data, using AMPAR antagonists, such as PER, might hinder synaptic potentiation and the effect of sleep on plasticity mechanisms in epileptic patients. However, this might not be the case. While it is true that antagonizing glutamate activity on AMPARs inhibits LTP in patients with epilepsy, this process is pathologic and might need to be counterbalanced. Indeed, both seizures and spikes induce LTP (172–176), which may compete with LTP occurring during everyday learning (177–179). Moreover, extracellular glutamate concentration was found to be higher during the dark period of the 24-h cycle in the hippocampus of both an MTLE translational animal model and control animals (180). However, glutamate levels had significant 24-h oscillations in epileptic hippocampi but not controls, possibly reflecting the circadian rhythmicity of temporal lobe epilepsies (180–182). Therefore, AMPAR antagonists may be useful in controlling glutamate’s circadian activity and inhibiting the dysfunctional LTP phenomenon, allowing the brain to learn an epileptogenic mechanism.

### 9.2. Sleep, epilepsy, and PER

Disrupted sleep, alteration of the sleep–wake pattern, and excessive daytime sleepiness are common complaints among patients with epilepsy and significantly impact the patient’s QoL (183, 184). The relationship between sleep and epilepsy is bidirectional, whereby factors, such as sleep deprivation and daytime sleepiness, may trigger seizures but may also be caused by epilepsy itself (185, 186). This complexity is compounded by the effects of ASMs, which may improve sleep (either directly or indirectly, e.g., by seizures and interictal spikes reduction) or through AEs that are detrimental to sleep (187, 188). Moreover, excessive daytime sleepiness, sleep apnea, insomnia, restless leg syndrome, and parasomnias are common in patients with epilepsy and can be overlooked when attributing tiredness to an unavoidable AE of an ASM (189).

Few clinical data are available on the relationship between PER and sleep in epileptic patients. The PERMIT study reported that around 10% of patients suffered somnolence as a treatment-related AE. However, objective studies have not confirmed these data. Preliminary data from various studies showed a subjective improvement in nocturnal sleep as measured by Pittsburgh Sleep Quality Index without excessive daytime somnolence (183, 189–193). The most recent review on the effects of ASMs on sleep architecture and daytime sleepiness in patients with epilepsy (183) evaluated five studies (Table 5) on PER and sleep. It concluded that PER had either no effect or improved sleep and that PER may be suited to patients with comorbid insomnia. Namely, PER does not affect sleep efficiency, total sleep time, sleep latency, REM sleep, N1 and N2 phases of NREM sleep, and arousal. PER appears to decrease wakefulness after sleep onset and improve the N3 phase of NREM sleep (193). Of note, in the study by Lee et al. (192), clinically diagnosed insomnia was less prevalent, less severe, and independent of depressive symptoms in the patients taking PER. Preliminary results from the AMPA trial, including 234 patients treated with adjunctive PER, suggest that PER does not negatively affect QoL or sleep up to 12 months of treatment (194).

**Table 5:** The effects of PER on various sleep parameters (184).

Study	Risk of bias	Main population (adult/adol/peds)	Comparator	SE	TST	SL	WASO	Arousals/awake	N1	N2	N3	REM	PSQI	ESS/SSS	Insomnia
González-Cuevas et al. (190)	High	Focal epilepsy (adult; >16 years)	Baseline	–	–	–	NA	NA	NA	NA	NA	NA	–	−/NA	NA
Lee et al. (192)	Moderate	Epilepsy (adult)	Drug-naïve	NA	NA	NA	NA	NA	NA	NA	NA	NA	NA	−/NA	↓^†^
Rocamora et al. (193)	Moderate	Refractory epilepsy (adult)	Baseline	↑	↑	↓	↓	↓	–	–	↑	–	–	−/NA	NA
Romigi et al. (191)	High	Focal epilepsy (adult)	Baseline	NA	NA	NA	NA	NA	NA	NA	NA	NA	↓	−/NA	NA
Toledo et al. (189)	Moderate	Refractory focal epilepsy (adult; >16 years)	Baseline	NA	NA	NA	NA	NA	NA	NA	NA	NA	↓ (3 months)/NA; − (6 months)/NA	− (3 months)/NA; ↓ (6 months)/NA	NA

Reproduced and adapted from Elsevier Ltd. under the terms of a Creative Commons license (CC BY-NC-ND 4.0). Adol, adolescent; ASM, anti-seizure medication; CAE, childhood absence epilepsy; EDS, excessive daytime sleepiness; ESS, Epworth Sleepiness Scale; HV, healthy volunteer; JME, juvenile myoclonic epilepsy; N1, stage N1; N2, stage N2; N3, stage N3; peds, pediatric; PBO, placebo; PSQI, Pittsburgh Sleep Quality Index; REM, rapid eye movement; SE, sleep efficiency; SL, sleep latency; SSS, Stanford Sleepiness Scale; TLE, temporal lobe epilepsy; TST, total sleep time; WASO, Wake After Sleep Onset; AND NA, not available.

↓, decrease; ↑, increase; −, no change.

† In a stepwise logistic regression model, clinically diagnosed insomnia was negatively associated with perampanel use in the study by Lee et al. (192).

One prospective observational study investigated the effectiveness of perampanel for the treatment of nocturnal seizures in 41 adult patients (30 available at 6-month follow-up). After 3 months, 66% were considered responders, and 51% were seizure-free. The number of monthly nocturnal seizures decreased significantly at 6 months (6.6 ± 0.4 vs. 10.6 ± 28.2), and subjective sleep disturbances improved at 3 months, suggesting that PER can be a suitable option in this setting (195). Another prospective study, although enrolling only 10 patients, found that PER treatment reduced daytime sleep propensity and had no negative effects on the sleep–wake cycle (196).

### 9.3. PER in sleep-related hyper motor epilepsy

Among specific epilepsy types closely related to or modulated by sleep and circadian rhythm, sleep-related hyper motor epilepsy (SHE) deserves particular attention. SHE is focal epilepsy characterized by seizures with complex hyperkinetic automatisms and/or tonic/dystonic asymmetric postures occurring mainly during sleep and arising mostly during NREM sleep (197). Only one study on PER and SHE has been published so far. Of the 14 patients with drug-resistant SHE, with a mean length of PER exposure of 24.6 ± 15.7 months, 10 were classified as responders, and six out of the 10 responders (60%) reported seizure-free periods of more than 6 months. The most common PER-associated AE was dizziness (25%), followed by malaise (10%). Therefore, PER might be considered an add-on ASM for patients with high drug refractory SHE. However, more evidence on a larger number of patients is needed to validate these results (198).

### 9.4. PER in sleep beyond epilepsy

The effects of PER on sleep might not be limited to epilepsy. The first polysomnographic study on PER outside epilepsy regarded restless legs syndrome (RLS) (199). Twelve of the 20 patients who completed the study (60%) were full responders (improvement in 50% of RLS severity score), and four (20%) partially responded. Furthermore, there were improvements in sleep stability as shown by increased total sleep time, sleep efficiency, N3 duration and percentage, reduced sleep latency, wake time after sleep onset, arousal index, N1 duration and percentage, REM sleep latency, periodic limb movement index, and arousal index. PER was well tolerated, with the adverse effects mainly being dizziness, drowsiness, headache, and irritability. The authors concluded that PER had significant therapeutic effects on both sensory and motor symptoms of RLS and might become a promising alternative to existing dopaminergic treatments in RLS (199).

In 2018, a small case series reported three patients with pharmaco-resistant insomnia treated with PER (200). The three patients described a significant improvement in their sleep pattern within a few days of treatment (dose of 4 mg at bedtime, titrating from 2 mg), without daytime sleepiness. An improvement in Pittsburgh Sleep Quality Index was observed after 1 month of treatment. In two patients, improvement persisted after 3 months of follow-up, while the other withdrew due to excessive irritability (200). In 2020, Abenza-Abildúa et al. (201) published the first study on patients with chronic resistant insomnia treated with PER. The observational study included 66 patients treated with one antidepressant or an anxiolytic drug, 33 of whom included in the exposed cohort were treated with adjunctive PER. In this cohort, 29 patients (88%) improved with PER combined with an anxiolytic or antidepressant drug. After 3 months of treatment, the number of hours of sleep gained was 2.5 h (range 1–5 h). The Insomnia Severity Index scale and the Pittsburgh scale both improved. The authors concluded that the inhibitory mechanism of PER on AMPARs seems to be responsible for the improvement in patients with chronic insomnia. To further validate these data, it would be necessary to perform a clinical trial compared with placebo (201) (Box 9).

BOX 9 **Sleep and epilepsy: conclusions and future directions.**

- AMPARs functions and LTP have a strict relationship with sleep.
- Seizures and interictal spikes may interfere with synaptic potentiation during everyday learning.
- PER does not alter cognitive functions (does not interfere with physiological sleep-related down-scaling processes?).
- PER seems to improve sleep quality and restless leg syndrome severity without inducing excessive daytime sleepiness.
- Future studies should be designed considering different types of epilepsy and comorbid sleep disorders.
- Polysomnographic studies should go into greater detail, investigate sleep microstructure, and be coupled with psychometric tests.
- Dosing PER at bedtime might be useful in improving overall sleep quality and, thus, daily somnolence.
- PER might be useful in treating SHE, including pharmacoresistant forms.

## 10. Migraine

### 10.1. The relationship between epilepsy and migraine

Headache and epilepsy are paroxysmal clinical disorders not infrequently occurring together in the same patient, with different interrelations. Increasing evidence points to a connection between seizures and migraine, which may include functional changes in membrane channels and neurotransmitters affecting cortical excitability. The imbalance between excitatory (glutamate) and inhibitory (GABA) factors appear to play a key role in both epilepsy and migraine (202). A study by Mainieri et al. (203) aimed to estimate the prevalence and clinical features of inter−/peri-ictal headaches in patients with epilepsy. Out of 388 patients, 54% reported a lifetime occurrence of headaches. The relationship between migraine and epilepsy is also evident in pediatric settings, as highlighted by Toldo et al. (204). Out of the 1,795 headache patients under 18 years consecutively diagnosed at their center, 3% had comorbidity between primary headache and idiopathic or cryptogenic epilepsy or unprovoked seizures. They also found that the prevalence of migraine in cases with epilepsy (82%) was significantly higher than in those without epilepsy (52%). On average, the risk of migraine in patients with epilepsy was 4.5-times higher than the risk of episodic tension-type headache (204). Since epilepsy and migraine are so intertwined, drugs that can treat both diseases also help discover their potential commonalities and differences (202).

### 10.2. PER in migraine and epilepsy

#### 10.2.1. Preclinical data

From this perspective, the use of AMPAR antagonists, such as PER, is starting to be investigated. The calcitonin gene-related peptide (CGRP) is expressed and released from the terminals of trigeminal neurons during a migraine attack. Its injection generates migraine pain, just as its pharmacological block has therapeutic effects (205). In acute rat brainstem explants, PER, given in the range of concentrations 0.01–100 μM, inhibited CGRP release in a concentration-dependent fashion compared with controls treated with a vehicle. The decrease was statistically significant from 10 μM onward (205). Therefore, PER might be useful in treating disorders related to inappropriate CGRP secretion, albeit additional preclinical data are needed.

#### 10.2.2. Clinical data

A phase II clinical study on PER and migraine was conducted in 2015. The primary outcome was the change from baseline in migraine period frequency per 28 days in the treatment phase. However, no difference with placebo was found after 23 weeks. Possibilities for failing the trial were the low dose (1.5–2 mg), lower than that normally used in epileptic settings and the slow titration phase. PER was initiated at a dose of 1.0 mg/day for the first 2 weeks, increased to 1.5 mg/day for the next 2 weeks, and then further increased to 2.0 mg/day for 10 weeks (206). An expert opinion published in 2017 (207) reviewed preclinical and clinical data on glutamate receptor antagonists with the potential for migraine treatment but did not include PER. The only AMPAR-selective antagonist was selurampanel, which did not achieve the primary efficacy criterion in the acute treatment of migraine in the only published clinical trial (208). However, the pain-free responses were comparable to those of sumatriptan, and the experimental drug provided relief from migraine in some subjects. In pharmacokinetic analyses, it was found that inter-subject variability was considerably higher, especially in the first 2 h, possibly indicating that the characteristics of early selurampanel absorption may have affected drug response (207, 208). The review’s authors concluded that, as clinical trial results conflicted with the preclinical data, glutamate did not play a decisive role after the attack had already been triggered. However, glutamate antagonists may be useful for migraine prophylaxis. Despite these not exceptional results, a recent study addressed the effectiveness of PER on epileptic seizures and migraine attacks in patients with epilepsy and comorbid migraine (95). In total, 31 patients were included, and 27 continued PER concomitantly with one (45%) or two ASMs (55%) at the 12-month follow-up visit. The mean PER dose at 6- and 12-month follow-up visits was 5.50 and 5.93 mg/day, respectively. A significant reduction was found not only in epileptic seizures but also in migraine attacks and monthly use of migraine rescue medications. Thus, the authors concluded that PER demonstrated good effectiveness, but future studies with possibly larger samples are needed to evaluate the efficacy of PER in migraine other than epilepsy (95) (Box 10).

BOX 10 **Migraine: conclusions and future directions.**

- AMPAR antagonism has been hypothesized as effective on migraine both with and without aura.
- The demonstrated effectiveness of PER on migraine in epileptic patients might depend on the amelioration of the epileptic condition.
- Further studies are required to evaluate the effectiveness of PER in migraine alone, possibly with higher doses than 2 mg, as studies on epilepsy and migraine suggest better effectiveness at higher doses.
- As migraine and epilepsy are more frequent in pediatric settings, this population requires interventional studies.

## 11. Overview of epilepsy and PER during pregnancy

Up to 0.7% of pregnant women have an epileptic syndrome (209) and frequently take ASMs during pregnancy to reduce the higher risk of seizure- and pregnancy-related complications compared to women without epilepsy (210–212). It is thus fundamental to investigate the safety of *in utero* ASM exposure to adequately balance the risk of adverse effects on fetal development with seizure control (210). Pregnancy results in enhanced drug elimination and decreased exposure; for example, reduced serum concentration for many ASMs have been reported. These changes in ASM pharmacokinetics must be considered as they might impact drug effectiveness and fetal development (213). Regarding PER, its exposure diminishes over time during pregnancy, resulting in 4- and 3-fold lower bound and unbound PER, respectively, at week 36 (214).

### 11.1. Preclinical data

Perampanel effects during pregnancy have been investigated in preclinical animal models. 1 mg/kg/day of PER (a clinically relevant dose as it is almost equivalent to 8 mg/day in humans) induced developmental toxicity in pregnant rats and rabbits (11). No PER-related effects on early embryonic development, developmental toxicity, fertility, and behavioral and reproductive development were found with doses of 1–30 mg/kg/day (214). On the other hand, PER could be linked with diverticulum of the intestine in pregnant rats (doses of 1–10 mg/kg/day) and post-implantation loss in rabbits and rats, especially at higher doses (30–60 mg/kg/day). PER also increased stillbirths and decreased the viability index at doses of 3 mg/kg/day already. Finally, PER (10/mg/kg/day doses) might delay some parameters of physical development, such as vaginal opening and preputial separation in females and males, respectively (214).

### 11.2. Clinical data

As PER is a relatively new drug, clinical data on pregnancy outcomes are scarce. For a comprehensive discussion, we refer to the work of Vazquez et al. (214), who collected data from the Eisai global safety database. They identified 96 pregnancies in PER-treated women, of which 71 with a known outcome. In 29% of women, no concomitant ASM use was recorded, but PER monotherapy could not be confirmed due to a lack of data. Forty-three pregnancies reached full term and resulted in normal live births, 18 were induced abortions, six spontaneous miscarriages, two incomplete spontaneous miscarriages, one premature delivery, and one stillbirth due to Fallot’s tetralogy. The reasons for induced abortions were generally unknown, but most were considered unrelated to PER. Six women had two PER-exposed pregnancies; one had two full-term normal births; three women had one full-term birth each, along with spontaneous abortion, incomplete spontaneous abortion, or an unknown outcome. Two women had both an induced abortion and a spontaneous abortion. Only five out of the 43 babies born at full-term pregnancy experienced adverse events. Of these, only poor sucking reflex and shallow breathing in one baby (concomitant clonazepam use) and low Apgar score in two babies (no concomitant ASMs) were considered possibly related to PER. No other fetal malformations nor birth defects were reported in babies who reached full term (214).

A more recent paper reports a case series of four PER-treated pregnant women, of which three received PER as an additional therapy throughout the entire gestation, and one started PER monotherapy at the 13th week of pregnancy. All pregnancies had favorable outcomes, and the newborns did not exhibit any major congenital malformations, low Apgar score, abnormal auxological parameters, or fetal pathology during follow-up. Although this suggests the administration of PER might be safe and tolerated by the patients and their fetuses, this report warrants caution and does not imply the general safety of PER during pregnancy as it enrolled a very low number of patients (215).

## 12. Overview of PER and psychiatric comorbidities in epilepsy

Approximately one-third of individuals with epilepsy have presented a psychiatric disorder at some point in their lives, with anxiety and depression being among the most common (20, 216). Psychiatric comorbidities significantly impact these patients’ QoL and are sometimes more impactful than seizures themselves. Moreover, it has been observed that individuals with epilepsy and psychiatric and behavioral comorbidities face a higher risk of premature death due to external causes (216, 217).

The most frequently reported PER treatment-emergent adverse events in phase III studies included irritability, somnolence, dizziness and fatigue, as well as anxiety, aggression, and anger when doses were ≥ 8 mg. The rates of other psychiatric conditions were similar to that of placebo (218, 219). However, RCTs have not always reported the patients’ psychiatric history or how the psychiatric assessments were carried out. In fact, PER might improve cognitive performances while not increasing irritability and aggression, even with long-term treatment and in pediatric populations (102, 103, 108, 220).

A prospective observational study enrolled 56 patients to specifically assess PER-related psychiatric symptoms through standardized scales over a 6-month observational period and showed no worsening in the patients’ QoL (221). PER did not increase depression and anxiety scores—even when scores indicated a mild level of anxiety before PER treatment. Although irritability was often reported in PER RCTs and observational studies, this study reports no increase in irritability, in line with the results of another smaller cohort (222). However, another study on 136 patients found that irritability and depression comorbidities before PER administration (as well as etiology of structural abnormalities and concomitant use of nitrazepam) were associated with psychiatric and behavioral deterioration after starting PER treatment (223). On the other hand, comorbidities such as insomnia, anxiety, and amnesia may improve after PER treatment, possibly resulting in the discontinuation of benzodiazepines seen in ∼25% of the enrolled patients. Moreover, as concomitant carbamazepine was positively associated with psychiatric improvements and negatively associated with the worsening of these symptoms, it is possible that carbamazepine alleviated the side effects of aggression and irritability caused by PER. However, it must be noted that this study had a retrospective design and was not based on standardized, validated scales for psychiatric assessment (223).

In conclusion, it seems that PER could either ameliorate or deteriorate psychiatric and behavioral symptoms according to the dose and titration schedule, patients’ characteristics at baseline, and concomitant ASM use. The available studies have some limitations, such as non-randomized, open-label designs, use of self-rated questionnaires or non-validated scales, and short follow-up. Well-designed studies using standardized scales are warranted to clarify the role of PER treatment in this setting.

## 13. PER beyond epilepsies: future perspectives

### 13.1. Stroke and neuroprotection

Acute ischemic stroke results from the abrupt interruption of focal cerebral blood flow (224). The resulting time-dependent cascade of the molecular events is characterized by decreased energy production, overstimulation of neuronal glutamate receptors (excitotoxicity), excessive intraneuronal accumulation of sodium, chloride, and calcium ions, mitochondrial injury, and cell death (224). Excitotoxicity and calcium overload are major factors contributing to the early stages of ischemic cell death. In fact, the glutamate overload leads to prolonged stimulation of AMPARs and NMDARs, dramatically enhancing the influx of calcium, sodium, and water into neurons. In turn, massive calcium influx activates catabolic processes (224).

#### 13.1.1. Preclinical data

Early experiments showed the neuroprotective effect of AMPAR antagonism in some animal models of neurodegeneration, stroke, and ischemia, especially in the initial phase after the damage (225–228). PER showed some neuroprotective effects in models of stroke/ischemia in preclinical settings (229). A study by Mazzocchetti and colleagues (230) investigated the effect of PER against *in vitro* ischemia. PER was able to avoid neuronal suffering even at low doses. It did not reduce basal excitatory synaptic transmission and did not alter LTP induction but blocked the pathologic ischemic LTP. This was probably due to PER’s capability to normalize the altered synaptic localization and function of AMPAR subunits occurring after an ischemic insult, as it restored the synaptic GluA1 subunit to control levels. In contrast, it did not affect GluA2 and GluA3 subunits (230). In other studies in different models of ischemia (231–233), PER significantly reduced infarct volumes, brain edema, neuronal apoptosis, microglial activation, pro-inflammatory cytokine expression (while increasing anti-inflammatory cytokines), oxidative stress, inducible nitric oxide synthase and neuronal nitric oxide synthase expression, and nitric oxide generation. It also suppressed neurodegeneration in the cortical ischemic boundary zone and improved cell viability, motor function, spatial working memory, and protection from ischemic stroke through claudin-5-mediated regulation of the blood–brain barrier permeability (231–233).

#### 13.1.2. Clinical data

Although AMPAR antagonists have shown neuroprotective efficacy in some pre-clinical settings, the successful completion of larger clinical trials has not been reported (228). Taken together, preclinical findings support the idea that PER is a potentially effective drug in counteracting ischemic damage and could effectively treat human stroke (229, 230). In this regard, well-designed clinical trials are needed. However, a study protocol for a trial of PER as an anti-epileptogenic treatment following acute stroke has recently been published (234). Meanwhile, an interventional study evaluating whether the prophylactic introduction of low-dose levetiracetam or PER in patients with moderate-to-severe middle cerebral artery infarct could prevent the development of post-stroke epilepsy (235) is expected to be completed by 2023 (Box 11).

BOX 11 **Neuroprotection: conclusions and future directions.**

- Decreased energy production, overstimulation of neuronal glutamate receptors (excitotoxicity), and excessive calcium overload are major factors contributing to the early stages of ischemic cell death.
- Early experiments with AMPAR antagonists showed their neuroprotective effects.
- PER showed different interesting effects at molecular, cellular, tissue, and behavioral levels resulting in an overall neuroprotective effect.
- Clinical studies on the effect of PER in human stroke and ischemia are lacking and needed.

### 13.2. Autism

Autism spectrum disorder (ASD) is a neurodevelopmental disorder sustained by complex pathophysiological mechanisms (236). Despite the unknown etiologies of ASD, AMPAR dysfunction and trafficking are possible contributing mechanisms (237).

#### 13.2.1. Preclinical data

Data on PER in preclinical studies of autism are not available. However, other AMPAR antagonists have been used in this setting. One study investigated the use of the AMPAR antagonist NBQX in rats with hypoxia-induced neonatal seizures (238). The results suggest that acute treatment with AMPAR antagonist during the latent period immediately following neonatal hypoxia-induced seizures can modify the activation of mTOR induced by seizures, reduce the frequency of later-life seizures, and protect against CA3 mossy fiber sprouting and autistic-like social deficits development (238). Another study used two different models of ASD, with either decreased or increased glutamate receptor expression and transmission (*Cntnap2* KO mice and VPA mice, respectively) (239). ASD symptoms improved using an AMPAR positive-allosteric-modulator or an AMPAR antagonist, normalizing the aberrant excitatory transmissions in the respective animal models. These results suggest AMPAR-derived excitatory neural transmission changes can affect normal social behavior. In the future, modulation of AMPARs, including by antagonists, might represent a possible treatment of ASD-associated social behavior deficits and symptoms, depending on the etiology and the underlying excitatory mechanisms involved (239).

#### 13.2.2. Clinical data

One study assessed the usefulness of PER and the relationship between behavioral impairments and EEG characteristics in epilepsy patients with ASD (240). Out of 17 patients, 11 (64.7%) had >50% reduction in seizures and interictal epileptiform discharges. Of these 11 patients, five (46%) with seizure/EEG responses were considered behavioral responders (i.e., ≥50% reduction in scores of the Japanese manuals for the Aberrant Behavior Checklist). At 12 months after PER administration, mean Aberrant Behavior Checklist scores were significantly decreased. Patients with frontal interictal epileptiform discharges showed a significantly greater correlation between seizures/EEG and behavioral improvements. Moreover, two of the six non-responder patients were considered behavioral responders (240). Therefore, PER treatment might be useful in reducing seizures in some patients and interictal epileptiform discharges for ASD patients with intractable epilepsy. Moreover, these results also support the usefulness of PER in improving neuropsychiatric impairments (240).

### 13.3. Other conditions

#### 13.3.1. Malformations of cortical development

Focal cortical dysplasia (FCD) is one of the most common cortical malformations causing untreatable epilepsy. For this reason, a significant proportion of FCD patients will undergo surgery for seizure control. However, evidence suggests a role for glutamate in the refractory nature of FCD and a possible beneficial effect of AMPAR antagonism in this setting (241–243). A preclinical study with human FCD brain slices showed, for the first time, that using PER reduced the burst firing behavior in human FCD microcircuits, thus resulting in a potent antiepileptic action. These findings pose an interesting rationale for investigating PER in patients affected by refractory epilepsy due to FCD (244).

#### 13.3.2. Autoimmune encephalitis

Anti-AMPAR autoantibodies can cause encephalitis, which often presents with seizures, confusion, memory deficits, and psychosis. A recent case report evaluated the effect of PER on a patient with encephalitis characterized by the presence of anti-AMPAR antibodies against the GluR2 subunit (242). First treatments with the combination of levetiracetam, carbamazepine, clonazepam, immunosuppressive therapies, and monthly periodic intravenous immunoglobin for 5 months were not effective for focal seizures and only slightly improved his memory. However, a rapid reduction in seizures was observed at the beginning of PER treatment. PER might be useful in anti-AMPA receptor encephalitis-associated seizures due to its attenuation of hyperexcitability caused by glutamate and Ca^2+^-permeable GluA4 subunit of AMPA receptors (242).

## 14. Conclusion

Perampanel has shown efficacy and effectiveness in controlling many types of seizures within different epileptic syndromes, especially if used as an early treatment option, and can even ameliorate cognition. It has a good safety and tolerability profile, although care must be taken when considering the titration schedule and the patients’ (psychiatric) comorbidities. By acting as a highly selective, noncompetitive antagonist on the AMPARs—the major contributors of glutamate-induced excitatory neurotransmission—PER has a broad-spectrum activity with the potential to tackle several conditions of the central nervous system. Nonetheless, data are still very scarce, and preclinical and clinical studies are warranted to explore PER potential, especially in conditions unrelated to epilepsy.

## Author contributions

FP, CC, AL, SL, CL, MM, SM, LN, FO, AR, ER, and CB: study conception and design and manuscript editing. CC, CB, AL, SL, CL, MM, SM, LN, FO, AR, and ER: collection and interpretation of data. FP: manuscript drafting. All authors contributed to the article and approved the submitted version.

## Funding

Medical writing and editorial and linguistic assistance were provided by Polistudium SRL (Milan, Italy) and supported by Eisai Co., Ltd.

## Conflict of interest

CC has received research support, speaker honoraria, and travel expenses from Bial, Eisai Europe Limited, GW Pharma, Lusopharma, PIAM Pharma, and UCB Pharma. CB has received consulting fees or speaker honoraria from UCB Pharma, Eisai, GW Pharmaceuticals, Bial, and Lusopharma. AL has received consulting fees or speaker honoraria from UCB Pharma, Eisai, GW Pharmaceuticals, Bial, and Lusopharma. SL has received speaker or consultancy fees from Angelini Pharma, Eisai, GW Pharmaceuticals, and UCB Pharma, and has served on Angelini Pharma, Arvelle Therapeutics, BIAL, EISAI, and GW Pharmaceuticals advisory boards. SM has received speaker or consultancy fees from Eisai, GW Pharmaceuticals, and UCB Pharma and has served on advisory boards for Eisai, UCB and GW Pharmaceuticals. LN has received speaker or consultancy fees from Fidia Pharma, Eisai, and Roche. FO has received speaker or consultancy fees from Eisai, GW Pharmaceuticals, and Angelini Pharma; FP is a collaborator of Polistudium SRL and was compensated for medical writing in accordance with the Good Publication Practice 2022. AR has received consulting fees or speaker honoraria from Angelini Pharma, EISAI, Jazz, and Bioprojet. ER has received speaker fees or funding and has participated in advisory boards for Arvelle Therapeutics, Angelini, Eisai, Kolfarma, JAZZ pharmaceuticals, Pfizer, GW Pharmaceuticals, UCB, and Lundbeck.

The remaining authors declare that the research was conducted in the absence of any commercial or financial relationships that could be construed as a potential conflict of interest.
